# Profiling of MicroRNA in Human and Mouse ES and iPS Cells Reveals Overlapping but Distinct MicroRNA Expression Patterns

**DOI:** 10.1371/journal.pone.0073532

**Published:** 2013-09-23

**Authors:** Siti Razila Abdul Razak, Kazuko Ueno, Naoya Takayama, Naoki Nariai, Masao Nagasaki, Rika Saito, Hideto Koso, Chen-Yi Lai, Miyako Murakami, Koichiro Tsuji, Tatsuo Michiue, Hiromitsu Nakauchi, Makoto Otsu, Sumiko Watanabe

**Affiliations:** 1 Division of Molecular and Developmental Biology, Institute of Medical Science, University of Tokyo, Tokyo, Japan; 2 Division of Biomedical Information Analysis, Department of Integrative Genomics, Tohoku Medical Megabank Organization, Tohoku University, Sendai, Miyagi, Japan; 3 Division of Stem Cell Therapy, Institute of Medical Science, University of Tokyo, Tokyo, Japan; 4 Stem Cell Bank Center for Stem Cell Biology and Regenerative Medicine, Institute of Medical Science, University of Tokyo, Tokyo, Japan; 5 Division of Pediatric Hematology/Oncology, Research Hospital, Institute of Medical Science, University of Tokyo, Tokyo, Japan; 6 Department of Life Sciences, Graduate School of Arts and Science, University of Tokyo, Tokyo, Japan; University of Houston, United States of America

## Abstract

Using quantitative PCR-based miRNA arrays, we comprehensively analyzed the expression profiles of miRNAs in human and mouse embryonic stem (ES), induced pluripotent stem (iPS), and somatic cells. Immature pluripotent cells were purified using SSEA-1 or SSEA-4 and were used for miRNA profiling. Hierarchical clustering and consensus clustering by nonnegative matrix factorization showed two major clusters, human ES/iPS cells and other cell groups, as previously reported. Principal components analysis (PCA) to identify miRNAs that segregate in these two groups identified miR-187, 299-3p, 499-5p, 628-5p, and 888 as new miRNAs that specifically characterize human ES/iPS cells. Detailed direct comparisons of miRNA expression levels in human ES and iPS cells showed that several miRNAs included in the chromosome 19 miRNA cluster were more strongly expressed in iPS cells than in ES cells. Similar analysis was conducted with mouse ES/iPS cells and somatic cells, and several miRNAs that had not been reported to be expressed in mouse ES/iPS cells were suggested to be ES/iPS cell-specific miRNAs by PCA. Comparison of the average expression levels of miRNAs in ES/iPS cells in humans and mice showed quite similar expression patterns of human/mouse miRNAs. However, several mouse- or human-specific miRNAs are ranked as high expressers. Time course tracing of miRNA levels during embryoid body formation revealed drastic and different patterns of changes in their levels. In summary, our miRNA expression profiling encompassing human and mouse ES and iPS cells gave various perspectives in understanding the miRNA core regulatory networks regulating pluripotent cells characteristics.

## Introduction

Induced pluripotent stem cells (iPSCs) have been extensively studied in recent years since the groundbreaking discovery by a group from Kyoto University [1]. The iPSCs were first reprogrammed from mouse somatic cells with the introduction of four transcription factors: Oct3/4, Sox2, Klf-4, and c-Myc (OSKM) [1], [2]. Since then, many groups have focused on finding the right formulation for making iPS cells (iPSCs) that closely resemble embryonic stem cells (ESCs) and that satisfy all the standard definitions of pluripotency, including the ability to differentiate into multiple cell types, germline transmission, teratoma formation, and contribution to chimeras [3].

The iPSCs can be reprogrammed from various sources, and embryonic fibroblasts [1] in mice and skin fibroblasts [2] in humans are the preferable sources. Somatic cells can be reprogrammed through various methods, using retroviruses [1], lentiviruses [4], adenoviruses [5], and small RNAs [6]. Differences in the choice of somatic cells source and reprogramming method cause variation among iPSCs and ultimately have a huge impact on safety pertaining to cell therapy. Prior to that, many studies examined genome-wide patterns of iPSCs and ESCs in complex regulatory networks linking chromatin structure and gene expression programs [7], as well as mRNA and microRNA (miRNA) expression profiles [7], [8], to improve understanding of genomic and epigenomic networks underlying reprogramming, self-renewal, and cell fate decisions.

One regulatory factor that has received increasing attention is miRNAs, which have the ability to regulate many target genes and control gene expression through translational repression and degradation [9]. miRNAs are expressed at different levels in a wide range of cells, including ESCs [10]–[12], iPSCs [13], and somatic cells [13]. Recent work showed that introduction of miR-302/367 resulted in higher reprogramming efficiency compared to exogenous OSKM transcription factors [5], indicating the importance of miRNAs in modulating the transition of somatic cells to pluripotent cells. In addition, miRNAs have been identified as important regulators of cell growth and differentiation and have also been used in the identification or classification of specific cell types [14]. In ES and iPS cells, several stem cell-specific miRNAs were identified and shown to be highly related to each other as they are grouped in a cluster on the same chromosome and are transcribed as a single primary transcript. The miRNAs, reported in numerous studies and expressed abundantly in human and mouse pluripotent cells, are members of the miR-302 cluster [12], [13], [15], [16]. Other previously identified miRNAs are a chromosome 19 microRNA cluster (C19MC) including miR-517a, miR-519b, miR-520b, miR-520b, and miR-521, which were found to be highly expressed in human stem cells [10]; the miR-290 cluster was only detected at high levels in mouse stem cells [11].

Several technologies are available for miRNA profiling, and each of them may be better than others in terms of sensitivity, cost efficiency, sequence dependence, or avoidance of potential contamination from artifacts. The selection of techniques with different approaches and experimental settings may explain fundamental differences observed, especially when a variety of pluripotent and differentiated cell lines from different species are used. Thus, in our work, we take advantage of a miRNA array system that offers consistent settings to be applied to different types of cells in both humans and mice. Currently, no study has focused on miRNA expression profiling in human and mouse ES and iPS cells at the same time. In this study, we comprehensively analyzed miRNA expression patterns in both human and mouse cells.

## Materials and Methods

### Culture of pluripotent stem cells

Human ES and iPS cell lines were used in conformity with the guidelines for derivation and utilization of human embryonic stem cells outlined by the Ministry of Education, Culture, Sports, Science and Technology, Japan. All human pluripotent cell lines used in this work were previously published, and are listed in Table 1 with references. Human iPSCs were maintained as described previously with some modification [2]. Briefly, human iPSCs and two human ESCs (khES3, H1) were maintained on DMEM/F12 (Sigma) supplemented with 20% knockout serum replacement (Invitrogen), 1% MEM non essential amino acid (Invitrogen), 1% L-Glutamine (Invitrogen), 0.2% 2-mercaptoethanol (Invitrogen), 5 ng/ml basic-FGF (Upstate), and the culture medium was changed daily. For splitting cells, 0.05% Trypsin-EDTA (Sigma) was used with or without 10 µM Rock inhibitor (TOCRIS). Other human ESCs (HUES3, HUES8, and HUES10) were maintained under feeder free condition as previously described [17] with modification. Cells were dissociated using CTK solution (ReproCells). Nanog-ips38 was donated from Prof. Shinya Yamanaka. B6 iPS#1 and #3 were established from B6 mouse, and this study was approved by the Animal Care Committee of the Institute of Medical Science, University of Tokyo. Mouse iPSCs were maintained in iPS medium consisting of DMEM high glucose (GIBCO), 15% fetal calf serum (FCS, GIBCO), 2% HEPES buffer solution (Nacalai Tesque), 1% L-glutamine (Nacalai Tesque), 1% non-essential amino acid (GIBCO), penicillin/streptomycin, 1000 U/ml of Leukemia Inhibitory Factor (ESGRO, Chemicon), and 0.2% ß-mercaptoethanol (SIGMA). The cells were cultured on 0.1% gelatin-coated plates with monolayer of mitomycin-C treated mouse embryonic fibroblast (MEF). Mouse ESCs (ES_K3_B6 and ES_RED) were cultured in the same culture conditions as iPSCs while ES_CCE, which was donated by Dr. Shin-ichi Nishikawa, was cultured in the same culture medium but without the feeder cells.

**Table 1:** Cell lines used in this study.

*Sample name*	*Abbreviation*	*Reprogramming conditions*	*Cell source*	*Cell condition*	*Ref.*
***Undifferentiated human embryonic stem cells (hESCs)***					
HUES3	HUES3	-	female	P26-3-3 sen	[49]
HUES8	HUES8	-	female	P17-4-7, TELA	[49]
HUES10	HUES10	-	female	P12-13, TELA	[49]
KhES-3	khES3, khES3_RI	-	female	+/− RI	
HI	H1	-	female	P57	[50]
***Undifferentiated human induced pluripotent stem cells (hiPSCs), all cells were sorted by SSEA-4***					
tkDA 3-4	DA, DA_RI	OSKM, retroV	adult dermal fibroblasts	+/− RI	[57]
TkCB 7-4	CB, CB_RI	OSKM, retroV	cord blood	+/− RI	[57]
tkCB Sev9	CB_sev9	OSKM, Sendai V	cord blood		[57]
tkPB Sev2	PB	OSKM, Sendai V	peripheral blood, CD34-rich		[57]
tkDN 4-M	DN	OSK, retroV	neonatal dermal fibroblasts		[57]
TkT 3 V1-7	TKT	OSK, retroV	peripheral blood T cells		[51]
H25 4 Sev	H254	OSKM, Sendai V	CD8+ T cell clone		[51]
***Embryoid bodies (EBs) and somatic cells***					
tkCB7-1 EBd7	EB_d7	-	Differentiated from tkCB7-1 iPS	-	-
tkCB7-1 EBd14	EB_d14	-		-	-
tkCB7-1 EBd21	EB_d21	-		-	-
HEK293	HEK293	-	human embryonic kidney 293	-	[52]
HeLa	HeLa	-	Adenocarcinoma cervix cell line	-	[53]
Y79	Y79	-	Retinoblastoma cell line	-	[18]
HDF Adult	HDF_AD	-	Dermal fibroblast cell	-	-
HDF Neonatal	HDF_N	-		-	-
PBMC_M	PBMC_M	-	Peripheral blood mono nuclear cells		
PBMC_T	PBMC_T	-			
tkDA3-4 NSC	DA_NSC	-	Neural stem cells from tkDA3-4 iPS	-	-
tkCB Sev9 NSC	CB_NSC	-	Neural stem cells from tkCB Sev9 iPS	-	-
***Undifferentiated mouse embryonic stem cells (miPSCs), all cells were sorted by SSEA-1***					
CCE	ES_CCE	-	129S6/SyEyTac		[54]
K3 B6	ES_K3_B6	-	129Sv x B6		
(2)RED2i	ES_RED	-	129S6/B6-F1		[55]
***Undifferentiated mouse induced pluripotent stem cells (miPSCs), all cells were sorted by SSEA-1***					
SP miPS	SP_iPS	OSKM, retroV	MEF (B6)		[57]
Nanog-ips 38	Nanog_iPS	OSKM, retroV	MEF (B6)	Sorted GFP+	
B6 iPS#1	B6_iPS_1	OSKM, retroV	MEF (B6)		
B6 iPS#3	B6_iPS_3	OSKM, retroV	MEF (B6)		
***Embryoid bodies and others***					
EB_ SP	EB_SP_iPS	-	SP miPS	Day 15 EBs	
EB_ Nanog 38	EB_Nanog_iPS	-	Nanog-38 ips	Day 15 EBs	
mouse embryonic fibroblast	MEF		ICR		[56]
tail tip fibroblast	TTF		ICR		

Abbreviation: OSKM; Oct3/4, Sox2, Klf4, c-Myc, retroV; retrovirus, sendai V; sendai virus, RI; Rock Inhibitor.

### Preparation of mouse embryonic fibroblasts (MEF) and tail-tip fibroblasts (TTF)

MEF and TTF were isolated and described in detail in previous paper [1]. Briefly, MEF were obtained by mincing the E12 embryo without internal organs followed by digestion in 0.05% trypsin-EDTA, and strained through 70 µm cell strainer (Falcon, BD Bioscience). Single cells obtained were cultured until confluence. TTF were obtained from adult tail tip by culturing tailbone that was cleaned by removing surrounding muscle and skin. The cleaned bones were then placed on the culture dish and medium was carefully added to the dish. The tail-tip was left undisturbed for two days before fresh medium were added. MEF and TTF were maintained in DMEM (Nacalai Tesque) supplemented with 10% FCS and 0.5% penicillin/streptomycin.

### Culture of cell lines

HeLa and HEK293 cells were maintained in DMEM (Nacalai Tesque) supplemented with 10% FCS and 0.5% penicillin/streptomycin. Y79 [18] was obtained from the Riken Cell Bank (identification number RCB1645) and maintained in RPMI1640 (Nacalai Tesque) supplemented with 10% FCS and penicillin/streptomycin. Peripheral blood mononuclear cells (PBMC) were prepared using a standard density gradient-separation technique from healthy adult volunteers after their documented informed consent was obtained. This study has been performed according to the Declaration of Helsinki, and the process involved has also been approved by the institutional review board (Institute of Medical Science, University of Tokyo Ethics Committee reference No. 20-8-0826). Participants provide their written informed consent to participate in the study. Human adult and fetal dermal cells (HDF_AD and HDF_N) were purchased from Cell Applications Inc. through Japanese trader TOYOBO and maintained in DMEM supplemented with 10% FBS, L-glutamine and 0.5% penicillin/streptomycin.

### Neural induction of human iPSCs

Neural induction was performed as described previously [19]. Briefly, human iPS cell cultures were dissociated using 0.25% trypsin, and plated on gelatin for 1 h at 37°C in the presence of Rock inhibitor (Y-27632, Wako) to remove MEF. The nonadherent iPSCs were plated on Matrigel (Becton, Dickinson and Company) coated dishes at a density of 10,000 cells/cm^2^ in MEF-conditioned iPS-medium supplemented with 10 ng/ml of bFGF (Peprotech) and Rock inhibitor. iPSCs were allowed to expand for 3 days, and the initial differentiation was induced by replacing media with knockout serum replacement media supplemented with 10 µM TGF-ß inhibitor (SB431542, Tocris) and 200 ng/ml of Noggin (R&D). From day 4, increasing amounts of N2/B27 medium (Neurobasal, 1% N2 supplement, 2% B27 supplement, 1% L-Gln, P/S) was added to the culture every 2 days (25%, 50%, 75%). Upon day 10 of differentiation, cells were passaged en bloc onto Matrigel-coated dishes in N2/B27 media supplemented with 10 ng/ml bFGF and 10 ng/ml EGF. The growing cells were dissociated and passaged every 7–10 days in the N2/B27 media supplemented with bFGF and EGF. To examine neurogenic potential of these cells, differentiation was induced by the removal of growth factors and the addition of FCS. Multi-lineage differentiation was confirmed by immunostaining of neuronal (ßIII tubulin) and astroglial (S-100ß) markers, thereby iPS-derived growing cells were defined as neural stem/progenitor cells (NSCs).

### Preparation of embryoid bodies (EBs)

EB formation of human iPSCs was carried out following previously reported procedures [20]. EBs were harvested at indicated time points. Mouse EBs were obtained by culturing iPSCs on a petri dish in the absence of leukemia inhibitory factor (LIF). Briefly, iPSCs were detached and collected cells were cultured for 30 minutes in a gelatin coated tissue culture dish to separate iPSCs from MEF feeder cells. Then, suspension cells were cultured as suspension in non-coated petri dishes. At day 7, 14, and 21 (human), or day 15 (mouse) of differentiation, cells were harvested, stained and sorted for SSEA-4 (human) or SSEA-1 (mouse) negative cells. Cells were all prepared under RNAs-free condition.

### Preparation of immature pluripotent cells for RNA extraction

The ES and iPS cells were thawed and cultured at appropriate density and were grown exponentially on 6-cm dishes containing pre-irradiated MEF feeder cells. On day 4 or 5, the cells were harvested with trypsinization and stained with anti-SSEA-4 (for human cells) or -SSEA-1 (for mouse cells) antibodies. Subsequently, the SSEA-4 or SSEA-1 positive cells (5×10^5^ cells/tube) were sorted by FACS (Moflo, Dako Cytomation) into collection tubes containing 200 µl of 2.5% FCS in PBS. Cells were immediately collected as pellets by centrifugation, and snap frozen in liquid nitrogen, and stored at −80°C until used.

### RNA extraction and miRNA examination

Total RNA was extracted according to the manufacturer protocol using miRVana miRNA isolation kit (Ambion). In some cases as noted in the manuscript, cells were undergone purification of total RNA containing small RNAs using RNeasy Plus Micro Kit (Qiagen). RNA (500 ng) was reverse transcribed using Taqman MicroRNA Reverse Transcription Kit (Applied Biosystems) and Megaplex RT primers Human Pool A or Rodent Pool A (Applied Biosystems). Then, cDNA was mixed with EagleTaq Master Mix with Rox (ROCHE) and was dispensed into each port of the TaqMan human or rodent MicroRNA Array A card v2.0 (Applied Biosystems). Human Array A card contains primers for 381 miRNAs including 3 positive control miRNAs, and 1 negative control primer. Rodent Array A card contains 341 primers for mouse miRNAs including 5 positive control miRNAs, and 1 negative control primer. Each miRNA card was run for real-time PCR using ABI PRISM 7900 HT Sequence Detection System (Applied Biosystems). The results were analyzed with SDS 2.4 and RQmanager 1.2.1 software (Applied Biosystems). All results except for Fig. S2 in File S1 are that of one representative result of each cell.

### Data Analysis

Hierarchical clustering (HC), nonnegative matrix factorization (NMF) and principal component analysis (PCA) were done using MeV 4.8.1 (Multi Experiment Viewer) software (http://www.tm4.org/mev/) according to the manufacturer's instruction. The miRNAs expression of the samples were clustered using average hierarchical clustering using Euculidean distance. Average value of distance of each miRNA was used to measure the cluster-to-cluster distance. NMF [21] was computed on miRNA expression profiles. We determined the minimum number of metaprofiles necessary to separate pluripotent cells from differentiated cells. PCA [22] was carried out on all genes under investigation to determine expression trends within the samples. A sample trend is shown in a scatter plot of the principal components PC1, PC2 and PC3.

## Results

### miRNA Expression Profiling of Pluripotent Stem Cells Using Quantitative PCR Arrays

To perform comprehensive profile miRNA expression patterns in human pluripotent stem cells, we used six ES cell lines, nine iPS cell lines, EBs at three different time points from one iPS cell line, two iPS cell-derived NSCs, and four primary tissues. Three cancer cell lines were also used as control somatic cells. As mouse samples, we profiled three ES cell lines, four iPS cell lines, two iPS-derived EBs, MEF, and TTF. The human iPSCs were generated by three or four reprogramming factors from different cell sources with different delivery methods (Table 1). Human arrays (377 miRNAs, excluding controls) and mouse arrays (335 miRNAs, excluding controls) were purchased from the same company (Applied Biosystems), but their probe sets were not exactly the same (Table S1 in File S2).

Before starting to analyze all samples, we first optimized a protocol to prepare cells to purify RNA for accurate examination of miRNA expression patterns. We examined whether we needed to purify iPSCs from feeder cells. We used MEF as feeder cells for both human and mouse iPSCs unless stated otherwise. We prepared RNA from iPSCs harvested with MEF, or from iPSCs purified by a cell sorter using an SSEA-4 antibody, and examined miRNA expression profiles using the miRNA array. We found that they gave different values for miRNAs, with the purified samples giving much lower Ct values for almost all miRNAs (Fig. S1A, B, C in File S1). Since primers for reverse transcription of human miRNAs may not cross with mouse sequences, we expected to obtain essentially the same results. However, our results indicated that RNA from feeder cells may reduce the sensitivity of detection, probably because the absolute amount of target cDNA is reduced by the cDNA derived from feeder cells. In addition, since mouse iPSCs should be separated from MEF, we decided to purify all human and mouse pluripotent cells using a cell sorter and SSEA-4 and SSEA-1 antibodies, and to then use them for RNA purification.

We then analyzed variation in the qPCR array data. We prepared three independent samples from tkCB7-4 cells and analyzed miRNA expression patterns using the human array. A summary of the results is shown in Fig. S2A in File S1. The results showed good reproducibility, especially for miRNAs expressed at relatively high levels. The lower panel in A shows 124 miRNAs with average ΔCt values <10, and 115 of which show standard deviations (SD) <1. The SD for nine miRNAs were >10, and raw data for these miRNAs are shown in Fig. S2B in File S1. In all cases, high SD values were caused by a false negative or false positive sample (Fig. S2B in File S1). Consequently, we decided to collect subsequent data in a single experiment.

**Figure 1:**
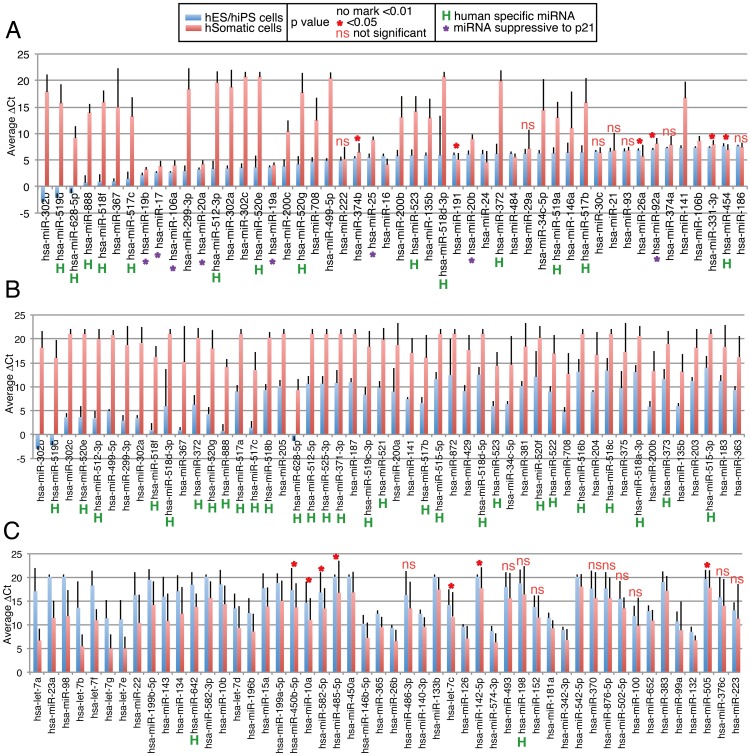
Comparison of expression level of miRNAs in human pluripotent stem cells and human somatic cells. Expression level of miRNAs in various cells was examined by qPCR based array, and ΔCt value was calculated as described. Average of ΔCt of immature ES/iPS (hES/hiPS-g, blue bar) and somatic cells and iPS derived NSCs (hSomatic-g, red bar) was calculated with standard deviation. A. Top 50 miRNAs from the highest expression level in ES/iPS are shown. miRNAs labeled with “H” are human specific miRNAs. B, C. Top 50 miRNAs from biggest difference of ΔCt between hES/hiPS-g and hSomatic-g are shown as well as values in hES/hiPS-g is bigger (B) and in hSomatic-g is bigger (C). In A–C. p value: non marked bars <0.01. * <0.05, ns not significant. Purple asterisk indicates miRNAs which have suppressive to p21 expression [28].

We first analyzed human samples. Raw quantitative PCR (qPCR) data were processed using RQ Manager (Applied Biosystems), and the resultant values were designated as Ct values (Table S2 in File S2). Then, variation of Ct values among samples was normalized by subtracting Ct values for mammalian U6, which was selected as an internal control because of its stable expression level, and the resulting values were designated as ΔCt values (Table S3 in File S2). All data were processed by this method. We then roughly compared the miRNA expression patterns of human pluripotent cells and other cells. Average ΔCt values for each miRNA in the human ES and iPS cell group (hES/hiPS-g) and the somatic cell, cancer cell, and NSC group (hSomatic-g) were compared, and statistically significant differences between the two groups were identified. We then listed the 50 miRNAs with the lowest average ΔCt values (highest expression levels) in hES/hiPS-g (Fig. 1A, blue bar) with the corresponding values in hSomatic-g (Fig. 1A, red bar). The list includes miRNAs that were previously reported to be highly expressed in ES or iPS cells, such as 302 cluster miRNAs [10], [12], [13], [15], [16], 17–92 cluster miRNAs [10], [13], and C19MC members [10], as expected, suggesting that our analysis worked reasonably well. More than half of the top 50 miRNAs in hES/hiPS-g showed higher expression levels than in hSomatic-g. p53 and its downstream effector p21 are induced during reprogramming, and minimizing the expression of both enhances iPS cell formation [23]–[27]. miRNAs that were reported to suppress p21 [28] are listed as miRNAs, but with similarly high levels of expression in hES/hiPS-g and hSomatic-g (Fig. 1A, purple asterisks). We then listed the top 50 miRNAs based on the largest differences in ΔCt values between hES/hiPS-g and hSomatic-g, as well as those with higher ΔCt values in hES/hiPS-g than in hSomatic-g (Fig. 1B). Notably, the list contains 23 members of the C19MC, which are human-specific miRNAs. In total, 28 human-specific miRNAs are on the list. In the array, 94 of 381 miRNAs are human-specific, suggesting that human-specific miRNAs are enriched in the highly expressed miRNAs in hES/hiPS-g. In contrast, when we listed miRNAs that have higher expression levels in hSomatic-g than in hES/hiPS-g, the list included only two primate-specific miRNAs (Fig. 1C). Members of the let-7 group, which are involved in developmental timing and expressed at higher levels in fibroblasts than in ESCs, are on the list (Fig. 1C), in accordance with a previous study [13].

### Analysis of Human miRNA Profiles

**Figure 2:**
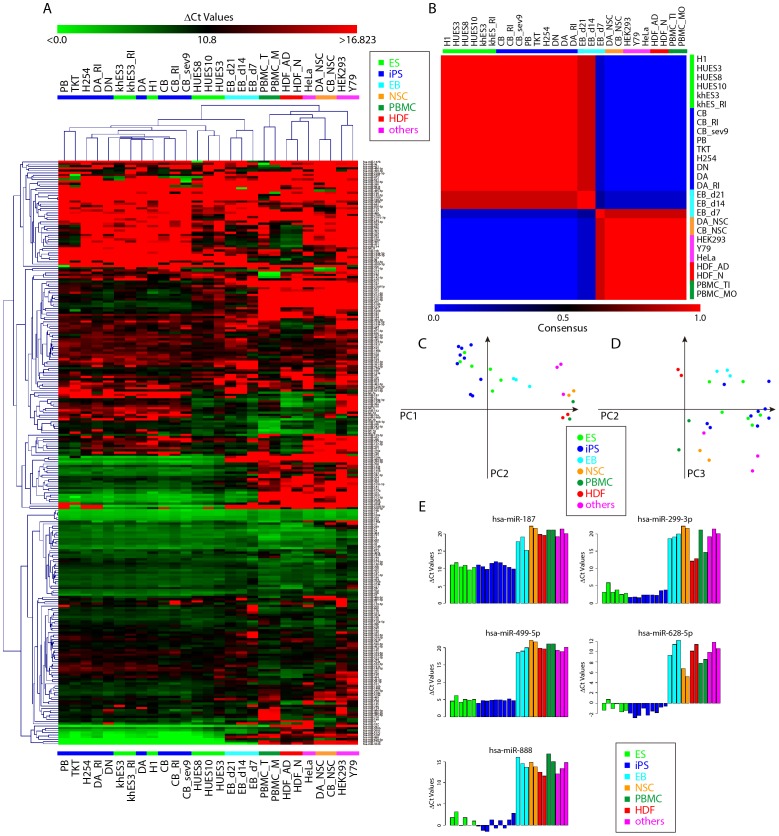
Clustering analyses of expression pattern of miRNA in human pluripotent cells, differentiated cells, and somatic cells. A. Comparison of relative expression levels of 263 miRNAs in human pluripotent cells, differentiated cells, and somatic cells. Red indicates low expression and green indicates high expression. B. Consensus clustering of cells by NMF with two metaprofiles. The NMF-transformed miRNA profiles distinguished two clusters. C, D. PCA was done using the same set of miRNA above. The first three components clustered cells (the first and second in C, the second and third in D) are shown. The first component accounts for 44.90%, the second for 8.83% and the third for 8.13% of the system variance. E. Expression levels of newly identified miRNAs are shown.

We next performed clustering analysis of miRNA expression patterns. Before the analysis, we excluded the 118 miRNAs with Ct values greater than 30 in all samples. The remaining 263 miRNAs were subjected to HC (Fig. 2A) and NMF (Fig. 2B) according to ΔCt values [21]. Both analyses showed clear segregation of ES/iPS cells from other cells (Fig. 2A, B). EBs were located closer to the ES/iPS cell cluster. In the somatic cells, two peripheral blood mononuclear cells (PBMC) and two dermal fibroblast cells were closely clustered. In the ES and iPS cell lines, three human ES cell lines were closely clustered, but clustering did not reflect differential clustering with categories of cell origin, methods, or use of ROCK inhibitor (Fig. 2A). We were interested whether iPS cells have some degree of similarity in miRNA expression pattern based on their origin; however using subsets of samples and/or miRNAs indicated no such relationship, suggesting that reprograming wipes out the characteristics of the original cells, at least in terms of miRNA expression patterns.

We then used PCA [29]. The first component showed clear segregation of ES/iPS and other cells (Fig. 2C). The second and third components segregated dermal fibroblasts and PBMCs (Fig. 2C, D). We then listed the first component eigenvectors (Fig. S3 in File S1). Eigenvectors with absolute values greater than 2 are shown in Table 2. Most of the listed miRNAs are members of the miR-302 cluster and two human-specific C19MC clusters (the miR-371/372/373 and miR-512∼ clusters), and these miRNAs were previously reported to be expressed in ES and iPS cells [10], [12]–[16], [30], [31]. Some of the miRNAs, such as miR-187, 299-3p, 499-5p, 628-5p, and 888, had not been reported to be characteristic miRNAs of ES or iPS cells in previous literature. miR-299-3p was described in ESCs [31], but not in iPSCs. However, a previous microarray study did not detect miR-299-3p in ES or iPS cells, perhaps because of the insensitivity of the microarray technique used [13]. Examination of the expression patterns of these miRNAs confirmed that they are strongly expressed in all ES/iPS cell lines, but not in other cells, including both primary cells and cancer cell lines (Fig. 2E). Similar to HC and NMF, PCA also showed no relationship between cell origin or method of establishing iPSCs and miRNA expression patterns. Note that miR-371-3p, 372, and 373 were highly expressed in H1 but not other ESCs (Fig. S4 in File S1). This tendency was also observed with H1, H7, and H9 cells in a previous report [14].

**Table 2:** Human miRNAs which have eigenvectors of the first component more than 2 in absolute value.

miRNA
hsa-miR-302a
hsa-miR-302b
hsa-miR-302c
hsa-miR-367
hsa-miR-372
hsa-miR-512-3p
hsa-miR-517a
hsa-miR-517c
hsa-miR-518b
hsa-miR-518f
hsa-miR-519d
hsa-miR-520e
hsa-miR-520g
hsa-miR-525-3p
hsa-miR-187
hsa-miR-299-3p
hsa-miR-499-5p
hsa-miR-628-5p

### Analysis of Mouse miRNA Profiles

**Figure 3:**
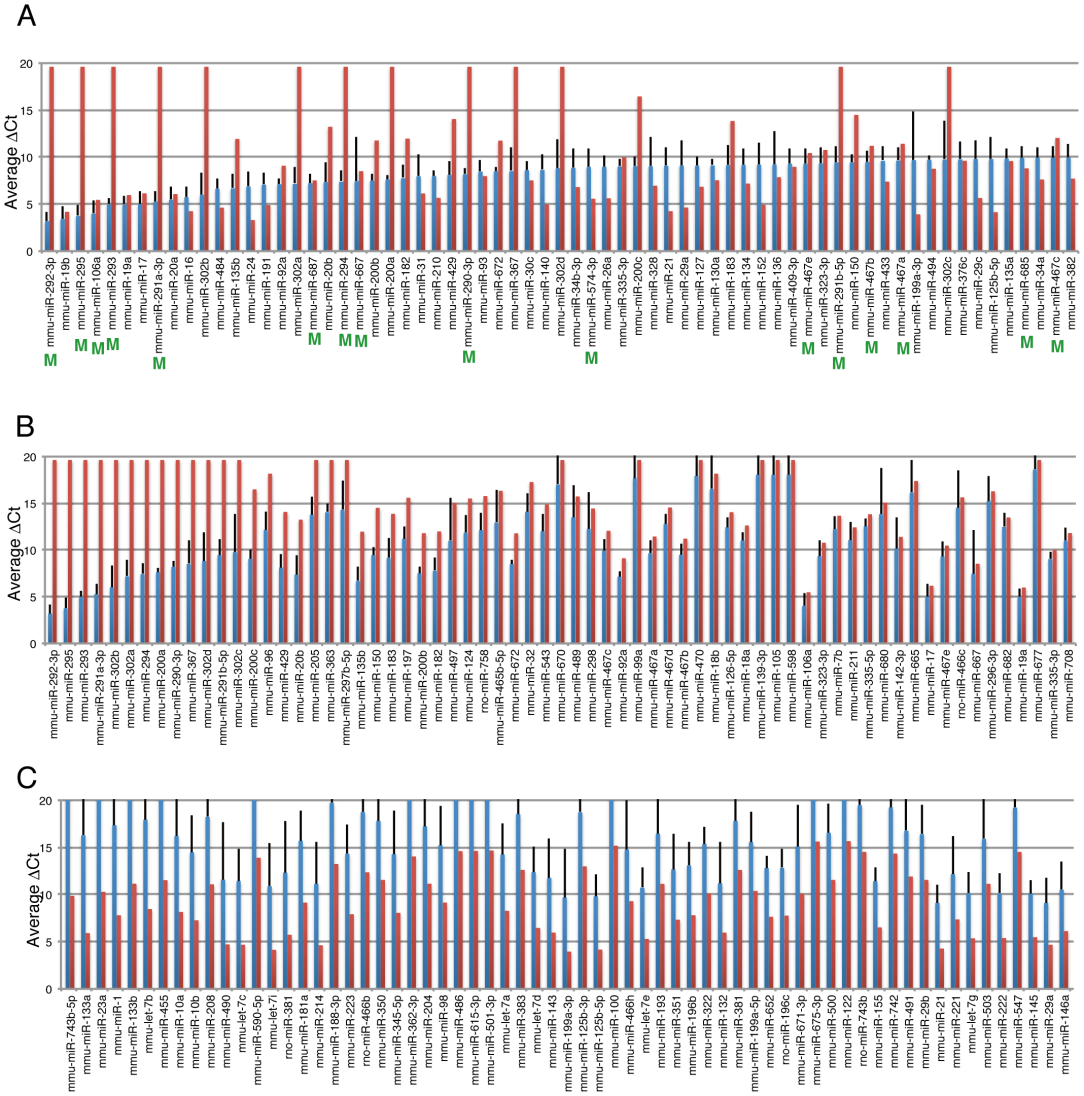
Comparison of expression level of miRNAs in mouse pluripotent stem cells and mouse somatic cells. Expression level of miRNAs in various cells was examined by qPCR based array, and ΔCt value was calculated as described. Average values with standard deviation of ΔCt of immature mouse ES/iPS are shown in blue bars. Average values of ΔCt of MEF and TTF are shown in red bars. A. Top 50 miRNAs from the highest expression level in ES/iPS are shown. miRNAs labeled with “M” are mouse specific miRNAs. B, C. Top 50 miRNAs from biggest difference of ΔCt between mES/miPS-g and mSomatic-g are shown as well as values in mES/miPS-g is bigger (B) and in mSomatic-g is bigger (C).

**Figure 4:**
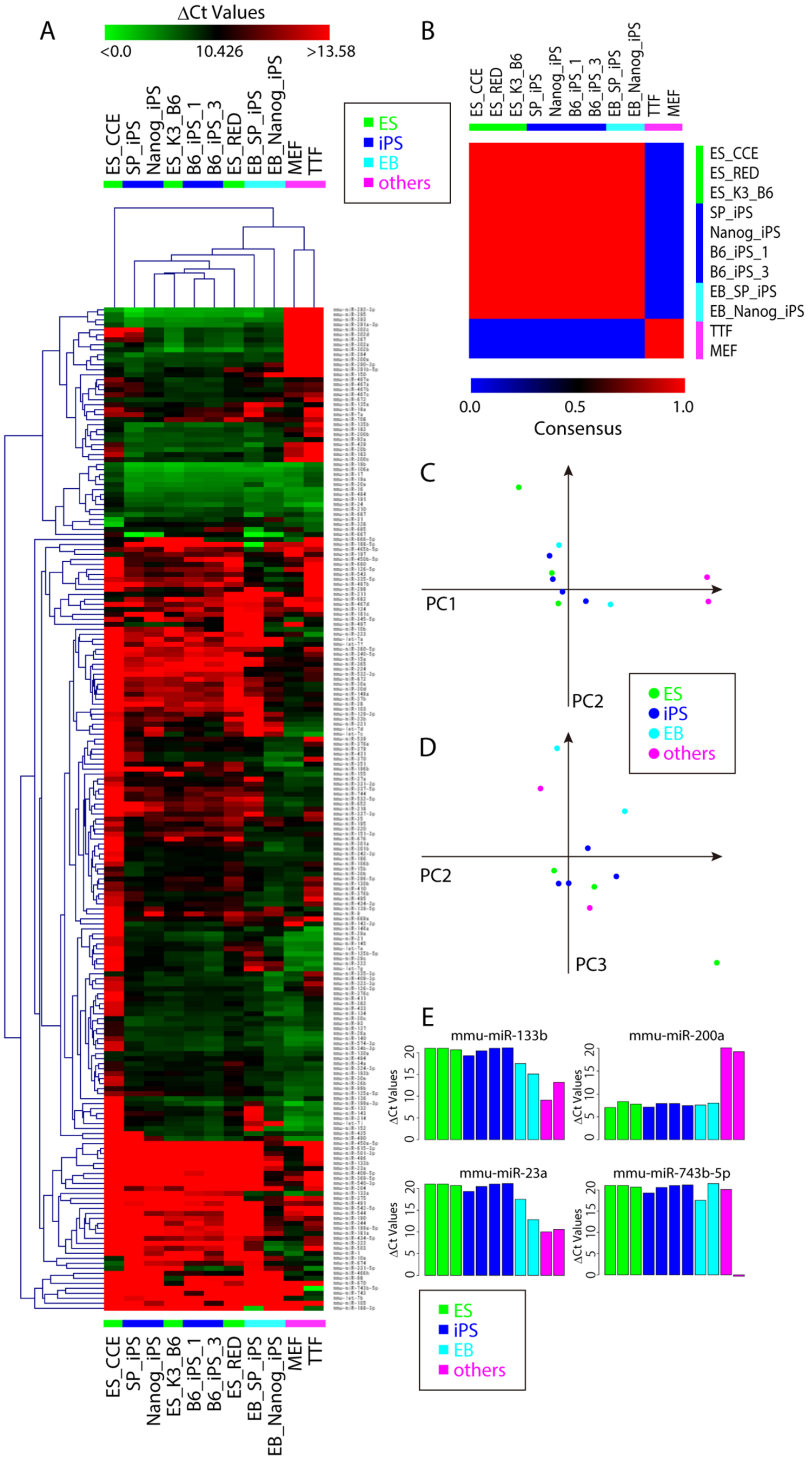
Clustering analyses of expression pattern of miRNA in mouse pluripotent cells, differentiated cells, and somatic cells. A. Comparison of relative expression levels of 201 miRNAs in mouse pluripotent cells, and somatic cells. Red indicates low expression and green indicates high expression. B. Consensus clustering of cells by NMF with two metaprofiles. The NMF-transformed miRNA profiles distinguished two clusters. C, D. PCA was done using the same set of miRNA above. The first three components clustered cells (the first and second in C, the second and third in D) are shown. The first component accounts for 41.99%, the second for 16.62% and the third for 11.08% of the system variance. E. Expression levels of newly identified miRNAs are shown.

We next analyzed the expression patterns of mouse ES and iPS cells, and other mouse cells. As in the human analysis, the Ct value of each miRNA (Table S4 in File S2) was subtracted from that of mammalian U6 (ΔCt, Table S5 in File S2). Simple comparison of the average ΔCt values of ES/iPS cells with the average ΔCt values of MEF and TTF showed that members of the 290 and 302 clusters are more highly expressed in ES/iPS cells than in MEF/TTF (Fig. 3A, B). miRNAs that are more highly expressed in MEF/TTF are listed in Fig. 3C. Members of let-7 (a, b, c, d, e), and 11 other miRNAs (miR-100, -10a, -10b, -132, -143, -181a, -196b, -199a-5p, -23a, -383, -505) are also listed as miRNA that are expressed at high level in somatic cells in human analysis (Fig. 1C), suggesting that these miRNAs are relatively low expression level in pluripotent cells commonly in human and mouse. For clustering analysis, miRNAs with ΔCt values greater than 30 in all samples were excluded. The remaining 201 miRNAs (out of 335 miRNAs) were further processed for clustering analysis. Clustering by HC and NMF showed clear separation of ES/iPS cells, except for ES_CCE cells, from MEF/TTF (Fig. 4A, B). PCA also showed good separation, and the first component contributed significantly to this separation (Fig. 4C, D). However, again like in human cell analysis, these methods failed to segregate iPS and ES to different categories. Eigenvectors of the first component with absolute values greater than 2 (Fig. S4 in File S1) are listed in Table 3. Among the listed miRNAs, the 290 and 302 clusters are well documented as ES and/or iPS cell-specific miRNAs [32]; miR-133b, 200a, 23a, and 743b-5p had not been characterized as ES and/or iPS cell-specific miRNAs. The ΔCt values of these miRNAs showed that expression of miR-133b and 23a was lower in ES/iPS cells than in MEF/TTF (Fig. 4E). miR-200a showed much higher expression in ES/iPS cells than in MEF/TTF (Fig. 4E). In contrast, miR-743-5p showed no difference, probably because this miRNA had an extremely low value in TTF. All other samples showed similar ΔCt values.

**Table 3:** Mouse miRNAs which have eigenvectors of the first component more than 2 in absolute value.

miRNA
mmu-miR-290-3p
mmu-miR-291a-3p
mmu-miR-291b-5p
mmu-miR-292-3p
mmu-miR-293
mmu-miR-294
mmu-miR-295
mmu-miR-302a
mmu-miR-302b
mmu-miR-302c
mmu-miR-302d
mmu-miR-367
mmu-miR-133b
mmu-miR-200a
mmu-miR-23a
mmu-miR-743b-5p

### Identification of miRNAs Distinguishing ES and iPS Cells

**Figure 5:**
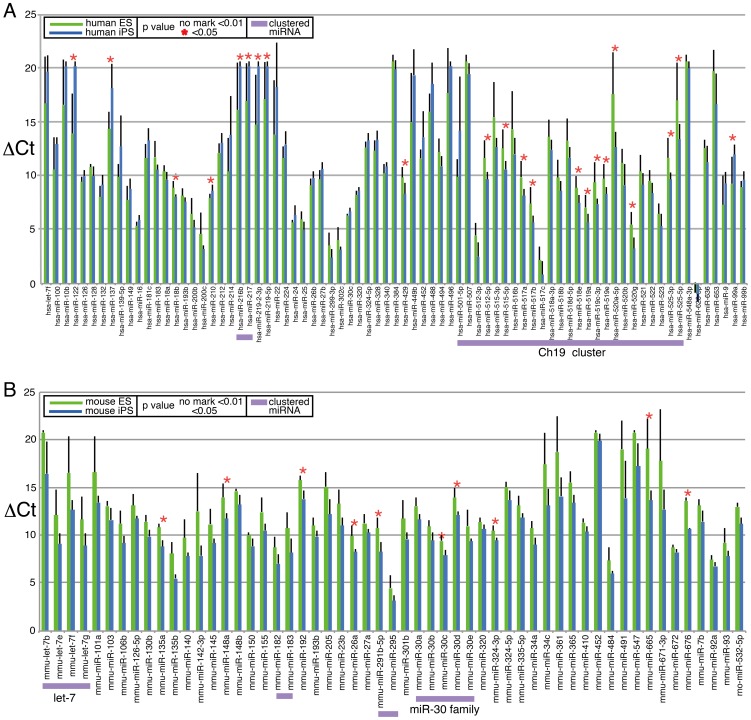
Comparison of miRNA expression levels of ES and iPS cells. A, B. Average ΔCt of human (A) or mouse (B) ES and human (A) or mouse (B) iPS are shown. miRNAs that have less than 0.05 p value between ES and iPS cells are listed by their miRNA number. Clustered miRNAs are indicated by violet under bar. p value: non marked bars <0.01. *(red asterisk) <0.05.

ES and iPS cells are distinguished by gene expression signatures [8], and we next questioned whether miRNAs exist that can distinguish human ES and iPS cells. We conducted HC and NMF analyses using ΔCt values for ES and iPS cells under several different sets of conditions, but HC or NMF did not separate ES and iPS cells. Moreover, PCA did not identify miRNAs that clearly distinguish human ES and iPS cells (data not shown). Therefore, we performed a simple comparison of average ΔCt values for ES and iPS cells, and miRNAs for which there were statistically significant differences between these two groups are shown in Figure 5A. Several miRNAs showed differences in expression between ES and iPS cells. Strikingly, most C19MC members showed higher expression in iPSCs than in ESCs (Fig. 5A, Fig. S5 in File S1). This result contradicts a previous study [13], which showed that C19MC miRNAs were more highly expressed in ESCs than in iPSCs. In that study, single type of ES cell line and single type of iPS cell line were compared, and we guess that the results may only be true for that particular case. Wilson et al. also reported that miR-886-5p expression was higher in iPSCs than in ESCs, but our results show that the average ΔCt values for miR-886-5p are identical in iPSCs (9.2, S.D. 2.8) and ESCs (9.2, S.D. 1.7). Study by Chi et al. listed 16 miRNAs that are differentially expressed in human ES and iPS cells [8], but only three of them showed similar trends in our study. Chin et al. used oligo DNA-based arrays (Ohio State University Comprehensive Cancer Center), and we cannot deny that different miRNA analysis protocols gave different results. Comparison of mouse ES and iPS cells identified several miRNAs that are expressed at significantly different levels in ES and iPS cells, and members of the let-7 and miR-30 families are more highly expressed in iPSCs than in ESCs (Fig. 5A).

### Comparison of Human and Mouse ES/iPS Cell-Specific miRNAs

**Figure 6:**
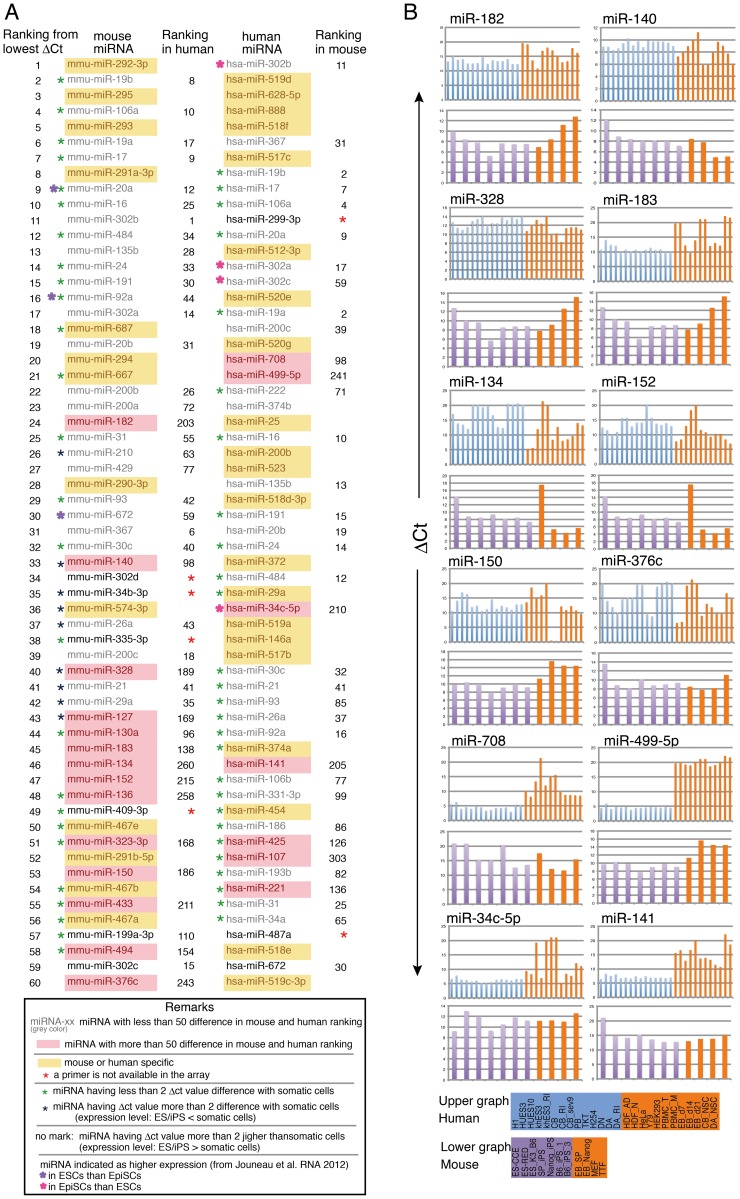
Comparison of miRNAs between human and mouse pluripotent stem cells. A. Top 60 miRNAs from lowest average ΔCt value of human and mouse pluripotent stem cells are listed. Ranking number in human and mouse are compared, and additional information are as indicated in the panel below of the figure. B. Expression levels (ΔCt) of miRNAs which are differentially expressed in human and mouse ES/iPS. Listed miRNAs are labeled with pink color but not with green asterisk in the panel A, which means miRNAs with more than 50 difference in mouse and human ranking and at the same time, having more than 2ΔCt value difference with somatic cells.

Human and mouse ES/iPS cells are in different states of pluripotency. Specifically, mouse ES/iPS cells are in naïve pluripotency and human ES/iPS cells are in the primed state [33], [34]. Mice have primed pluripotent stem cells called epiblast stem cells [4], [35], and differences in gene expression patterns have been reported. We compared the miRNAs with the highest expression (lowest ΔCt values) in human and mouse ES/iPS cells. Figure 6A shows the top 60 miRNAs in mouse ES/iPS and human ES/iPS cells. Green asterisks indicate housekeeping miRNAs, since differences in the ΔCt values between ES/iPS-g and Somatic-g are less than 2 (Fig. 1), indicating that the expression level was not very different between the groups. miRNAs with differences in rank of greater than 50 are highlighted as pink. Expression levels (ΔCt) of miRNAs which have more than 50 difference in mouse and human ranking and have more than 2ΔCt value difference with somatic cells are shown in Fig. 6B. Most of the top-ranked miRNAs specific for ES/iPS cells are either mouse-specific or human-specific. In the mouse list (Fig. 6A), six of the top eight mouse-specific miRNAs are members of the miR-290 cluster. The human miR-371 cluster was predicted to exist based on sequence similarity to members of the mouse miR-290 cluster [36], [37]; indeed, one of the members of the miR-371 cluster, miR-372, is 33^rd^ in the human miRNA list (Fig. 6A). C19MC originated evolutionarily from the miR-371-373 cluster [38], and some C19MC members share the same seed sequence [39]. Six of the top eight human-specific miRNAs are C19MC members. The seed sequence of miR-302 is identical to those of miR-372, miR-373 [39], [40], and some C19MC members. Members of the miR-302 cluster were ranked 11^th^, 17^th^, 34^th^, and 59^th^ in the mouse list, and 1^st^, 30^th^, and 33^rd^ in the human list. In addition, miR-367, which is a distantly related member of the miR-302 cluster [36], was 37^th^ in the mouse list and 17^th^ in the human list. Therefore, large numbers of miRNAs that are orthologs or have an identical seed sequence are among the most abundant miRNAs in both mice and humans. This suggests redundancy of these abundant miRNAs. In fact, miR-302 and miR-372 have been shown to promote reprogramming, and miR-302 and miR-372 inhibit the TGF-ß-induced epithelial–mesenchymal transition, as does mouse miR-294 [37]. In addition, other miRNAs—such as miR-17, -20a, -93, -106b, -106a, and -20b, and miR-302 members—which share the same sequence as the miR290 cluster, the miR-371 cluster, and C19MC, and were detected at high levels in both human and mouse ES/iPS cells, were shown to mediate reprogramming by targeting Tgfßr2 and p21 during the mesenchymal-to-epithelial transition during the initiation stage of reprogramming [6].

miR-182, 708, and 499-5p showed large difference of expression between human iPS/ES and somatic cells but relatively mild difference in mouse case (Fig. 6B). However, previous literature did not explain the specific expression of these miRNAs in human and mouse pluripotent cells.

### Changing Levels of miRNAs when iPSCs Differentiated

**Figure 7:**
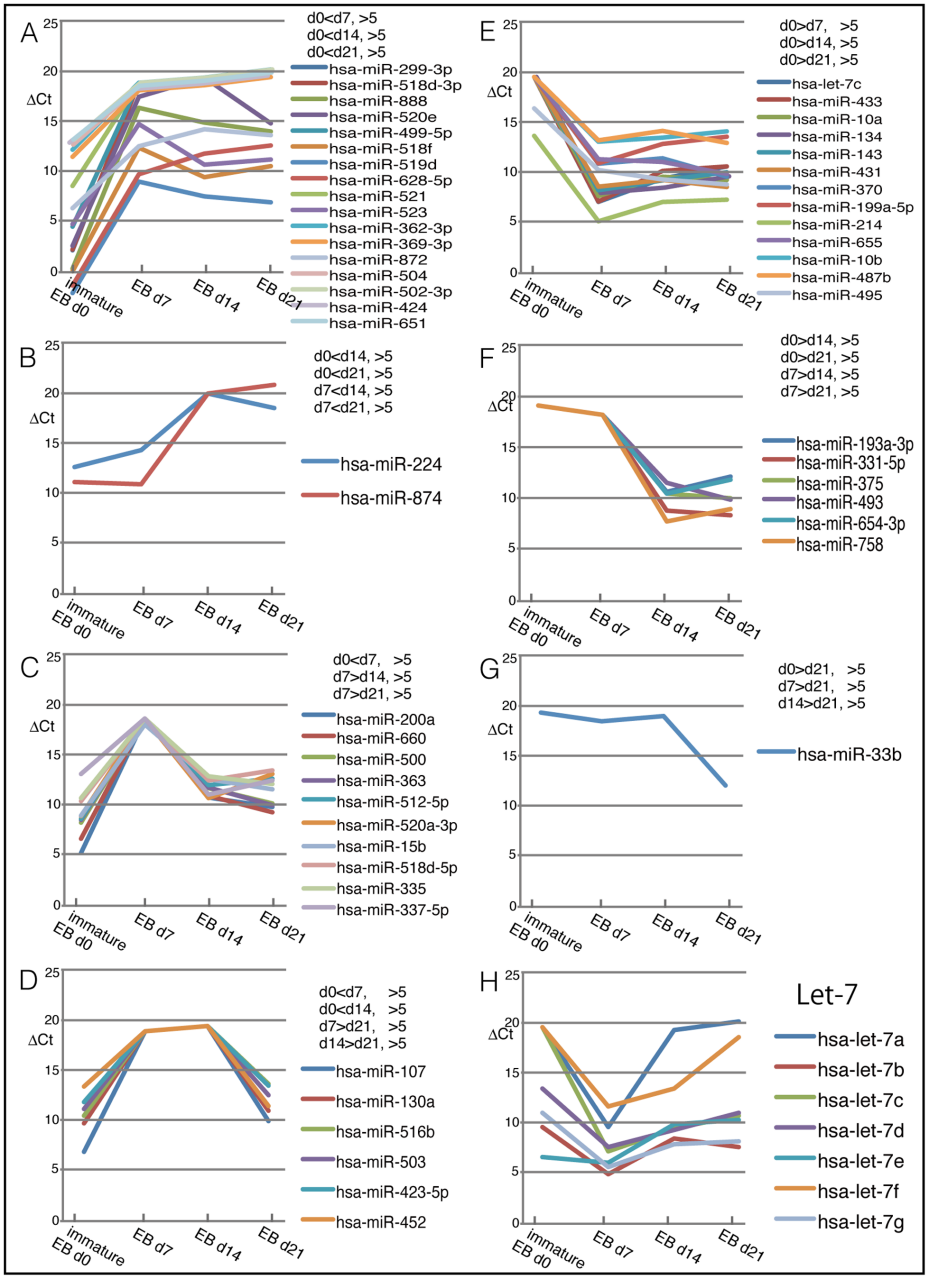
Alteration of level of miRNAs during formation of human EB. Human EB was formed, and expression profile of miRNAs was examined at day 7, 14, and 21 after starting formation of EB. Levels of miRNAs were categorized by patterns of changing of expression (ΔCt values).

We next examined the expression levels of miRNAs when iPSCs differentiated. Human EBs were formed from human iPS cells and harvested at three time points. Then, changes in miRNA levels were examined (Fig. 7). Expression levels of most of the miRNA were drastically changed after EB formation, and we categorized the miRNAs into seven groups according to their expression patterns (Fig. 7A–G). The patterns were divided into two groups: downregulated (Fig. 7A–D) and upregulated (Fig. 7E–G) after EB formation. Figure 7A shows miRNAs that were expressed highly in human ES/iPS cells (denoted by immature EBs, d 0) and at low levels in EBs at all the examined time points (days 7, 14, and 21). Our newly identified ES/iPS cell-specific miRNAs, miR-299, -499-5p, -628-5p, and -888, were included in the list, further confirming that these miRNAs are more highly expressed in immature cells than in differentiating or differentiated cells. However, some miRNAs that are highly expressed in immature EBs show reduced expression in early differentiation and a decrease when the cells undergo further differentiation at days 14 and 21 (Fig. 7B). The miRNAs categorized in Figure 7C and 7D may not be necessary or should not be expressed at the early and middle stage of differentiation, as shown by their low expression levels on days 7 (Fig. 7C) or days 7/14 (Fig. 7D) of differentiation, but high or increased expression on day 14 and 21. The let-7 miRNAs, which were reported to regulate cell differentiation, were characterized (Fig. 7H). They were mostly upregulated by EB formation, as previously reported [31], [41], [42], but their levels decreased following further differentiation (Fig. 7E). There are several miRNAs which increased with differentiation with delayed time course (Fig. 7F, G). Differentiation of iPS/ES to multiple lineages of cells is sequential events, and role of the miRNAs in a particular developmental stage can be surmised from such fluctuation of expression level of miRNAs during differentiation. These expression patterns further support the idea that miRNAs are highly regulated in pluripotent cells and regulate cell differentiation.

## Discussion

miRNA has received much attention in recent years in basic and applied science. For pluripotent cells, miRNA is expected to be a powerful tool from technical aspects, in addition to biological interests. Profiling miRNA expression patterns in ES and iPS cells is critical. Several technologies are available for miRNA profiling, and each of them may be better than others in terms of sensitivity, cost efficiency, sequence dependence, or avoidance of potential contamination by artifacts. The selection of a technique with a different approach and experimental setting may explain fundamental differences observed, especially when a variety of pluripotent and differentiated cells from different species are used. Thus, in our work, we took advantage of a miRNA array system that offers a consistent setting when applied to different types of cells in both humans and mice. Thus far, no study had profiled miRNA expression in human and mouse ES and iPS cells at the same time. In this study, we comprehensively analyzed miRNA expression patterns in purified ES and iPS cells, and somatic cells, in both humans and mice. Since we attempted to establish a reliable database, we carefully examined the reproducibility of the results obtained by the qPCR-based method, and the effects of sample preparation on the quality of the results were also carefully examined. We found that RNA prepared from a mixture of iPSCs and feeder cells gave weaker signals than that from purified iPSCs. Clustering analysis of miRNA expression patterns of purified iPSCs or iPSCs plus MEF with those of EBs clearly separated iPSCs from EBs, while the iPSCs plus MEF mixture was much closer to EBs (data not shown). Therefore, we prepared all pluripotent cells by purification using a cell sorter and SSEA-4 (human) or SSEA-1 (mouse). The resulting comprehensive data allowed us to compare various different subsets of pluripotent cells, and we identified several miRNAs that had not previously been reported to characterize ES/iPS cells. Note that miR-628-5p and miR-888 are primate-specific miRNAs, which makes them very useful candidate miRNAs to distinguish not only pluripotent and differentiated cells, but also human and other non-primate species. Why could we find new miRNAs after numerous similar efforts? In the case of miR-187, 299-3p, 499-5p, 628-5p, and 888, these miRNAs showed nearly negligible values or were not examined in previous studies [13], [14]. The high and stable sensitivity of our analysis may explain the current results. We attempted to predict the functions of these miRNAs in iPS/ES cells in several ways. Seed sequence examination indicated no similarities to the known seed sequences of pluripotency-specific miRNAs such as AAGUGC in miR-302b-3p, miR-373, miR-520e, miR-519c-3p, miR-520a-3p, and miR-520b; AGUGCC in miR-515-3p and miR-519e; and AAGUG in miR-519d. Their potential target genes were identified using several public databases, including miRanda, miRDB, miRWalk, RNA22 and TargetScan (http://www.umm.uni-heidelberg.de/apps/zmf/mirwalk/index.html). The databases predicted various physiological functions for these miRNAs. However, we were unable to correlate these functions with characteristics specific to iPS/ES cells. Few previous reports of these miRNAs are available. However, the involvement of miR-187, miR-299-3p, and miR-628-5p in some aspects of biology, including cancer, has been reported [43]–[46]; thus these miRNAs may play roles in regulating the proliferation of iPS/ES cells.

Differences in miRNA expression patterns between ES and iPS cells were one of the focuses of the current study. Our clustering analysis failed to segregate ES and iPS cells. However, simple comparison of average values for human ES and iPS cells identified several miRNAs with statistically significant differences in expression between ES and iPS cells. Among them, C19MC members showed higher expression levels in iPSCs than in ESCs. C19MC harbors the largest cluster of miRNA genes that developed in a recent mammalian evolution [38], [47]. It spans a genomic region of about 100 kb, which contains 39 miRNAs. A common enhancer for C19MC miRNAs may contribute to differences in the expression levels between ES and iPS cells; however, mechanisms regulating C19MC miRNA transcription have not been well characterized. C19MC originated evolutionally from the miR-371-373 cluster, the human ortholog of the mouse 290 cluster [38]. However, the miR-371-373 cluster (humans and mice) and miR-290 cluster (mice) did not show significant differences in expression between iPS and ES. The presence of abundant miRNA with similar seed sequences in C19MC indicates the generation of novel miRNAs during primate evolution, which may have led to functional diversification [38]. Therefore, higher expression of C19MC members, but not human miR-371-373 or mouse miR-290 members, in iPSCs indicates that the acquired functions of C19MC members may contribute to the biological significance of different expression levels in ES and iPS cells. We are wary of concluding that the observed difference in miRNA expression between ESCs and iPSCs is consistent. Through examination of the SD values, we found that most miRNAs that show statistically significant differences between ESCs and iPSCs have relatively high SD values (Fig. 5). We analyzed large numbers of cells, and detected statistically significant differences in several miRNAs; however, whether these differences reflect the difference between ES and iPS cells should be examined carefully. A similar discussion was presented in a previous report of global chromatin structure and gene expression data of ESCs and iPSCs [7]. There was little difference between ESCs and iPSCs in terms of H3K4me3 and H3K27me3 marks [7]. Gene expression profiles confirmed that the transcriptional programs of ESCs and iPSCs show few consistent differences [7]. Although the materials examined differed, our data are similar, and so careful evaluation of the biological significance of differences in the expression levels of miRNAs is required.

By analyzing miRNAs in both human and mouse pluripotent cells, we sought to identify differences in miRNA expression patterns between humans and mice. We were concerned as to whether comparison of Ct values obtained using different primers (human and mouse) would give meaningful results, but the ranks of miRNAs were quite similar in humans and mice (Figs. 1, 3). This is probably because the qPCR conditions for each primer set were well adjusted to give proper Ct values, as the supplier claimed. Among common human and mouse miRNAs, not many miRNAs showed great differences in expression between humans and mice. Whether these miRNAs correlate with differences in pluripotency levels between humans and mice is an interesting issue. Previous work examining miRNA expression in naïve (ESC) and primed (epiblast stem cell, EpiSC) mouse pluripotent stem cells revealed that several distinct miRNAs are differentially expressed in ESCs and EpiSCs [48]. Hierarchical clustering of miRNA expression profiles in two ESC lines and three EpiSC lines showed that the three EpiSC samples clustered closely together and could be discriminated from the ESCs [48]. Among 987 miRNAs whose expression differed between ESCs and EpiSCs, 226 miRNAs were more highly expressed in ESCs, while 76 miRNAs were more highly expressed in EpiSCs [48]. However, among the most abundant differentially expressed miRNAs (19 miRNAs with higher expression in ESCs and 22 miRNAs with higher expression in EpiSCs), three and four miRNAs that are more highly expressed in ES and iPS cells, respectively, showed similar trends in our comparison of human and mouse miRNAs (Fig. 6). Further functional analysis of these miRNAs may improve understanding of the molecular mechanisms of the level of stem cell pluripotency.

## Supporting Information

File S1 **Figure S1. Expression pattern of miRNA of purified and un-purified iPS.** Expression of miRNA was examined by qPCR array usingcDNA prepared from purified iPS cells and un-purified iPS cells, which are mixture with feeder cells. Average values are average DCt values of all human iPS. **Figure S2. Expression pattern of miRNAs of purified human iPS, tkCB 7-4 was analyzed 3 times independently.** Lower panel in A is enlarged part of upper panel with name of miRNA. There are 9 miRNAs which have more than 5 SD value in all, and row data of 3 samples of these miRNAs are shown in B. **Figure S3. The eigenvectors of the first component of PCA of human cells. Figure S4. The eigenvectors of the first component of PCA of mouse cells. Figure S5. Relative expression values of members of C19MC miRNA.** Expression levels of members of C19MC miRNA of human ES (green bar) and human iPS (blue bar) are shown.
(PDF)

File S2 **Table S1. List of miRNAs in Array A (TaqMan Array Card) of mouse and human. Table S2. Ct values of human samples.** Raw quantitative PCR (qPCR) data were processed using RQ Manager (Applied Biosystems), and the resultant values were designated as Ct values. **Table S3.** Δ**Ct values of human samples.** Variation of Ct values among samples was normalized by subtracting Ct values for mammalian U6, which was selected as an internal control because of its stable expression level, and the resulting values were designated as ΔCt values. **Table S4. Ct values of mouse samples.** Raw quantitative PCR (qPCR) data were processed using RQ Manager (Applied Biosystems), and the resultant values were designated as Ct values. **Table S5. ΔCt values of mouse samples.** Variation of Ct values among samples was normalized by subtracting Ct values for mammalian U6, which was selected as an internal control because of its stable expression level, and the resulting values were designated as ΔCt values.
(XLSX)
