# Supplementary figures and images for: Profiling of MicroRNA in Human and Mouse ES and iPS Cells Reveals Overlapping but Distinct MicroRNA Expression Patterns
Source: PLoS One. 2013 Sep 23;8(9):e73532. doi: 10.1371/journal.pone.0073532 (PMC3781120; doi:10.1371/journal.pone.0073532)

Fig.S1 Siti et al.

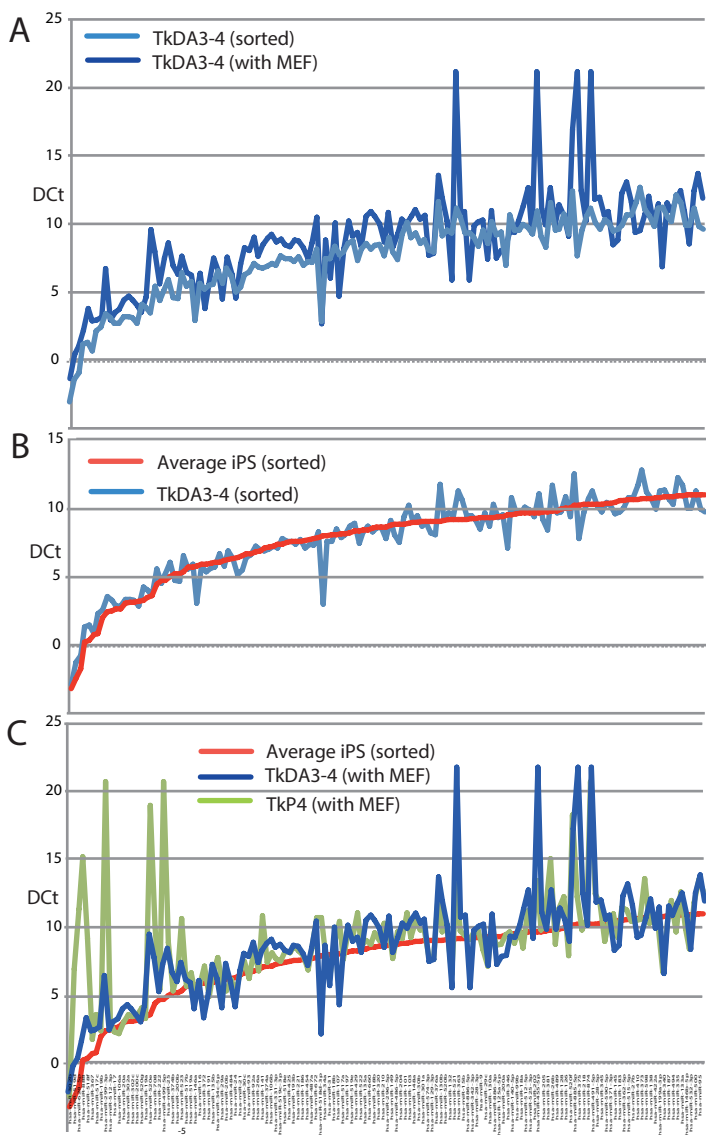

Fig.S2 Siti et al.

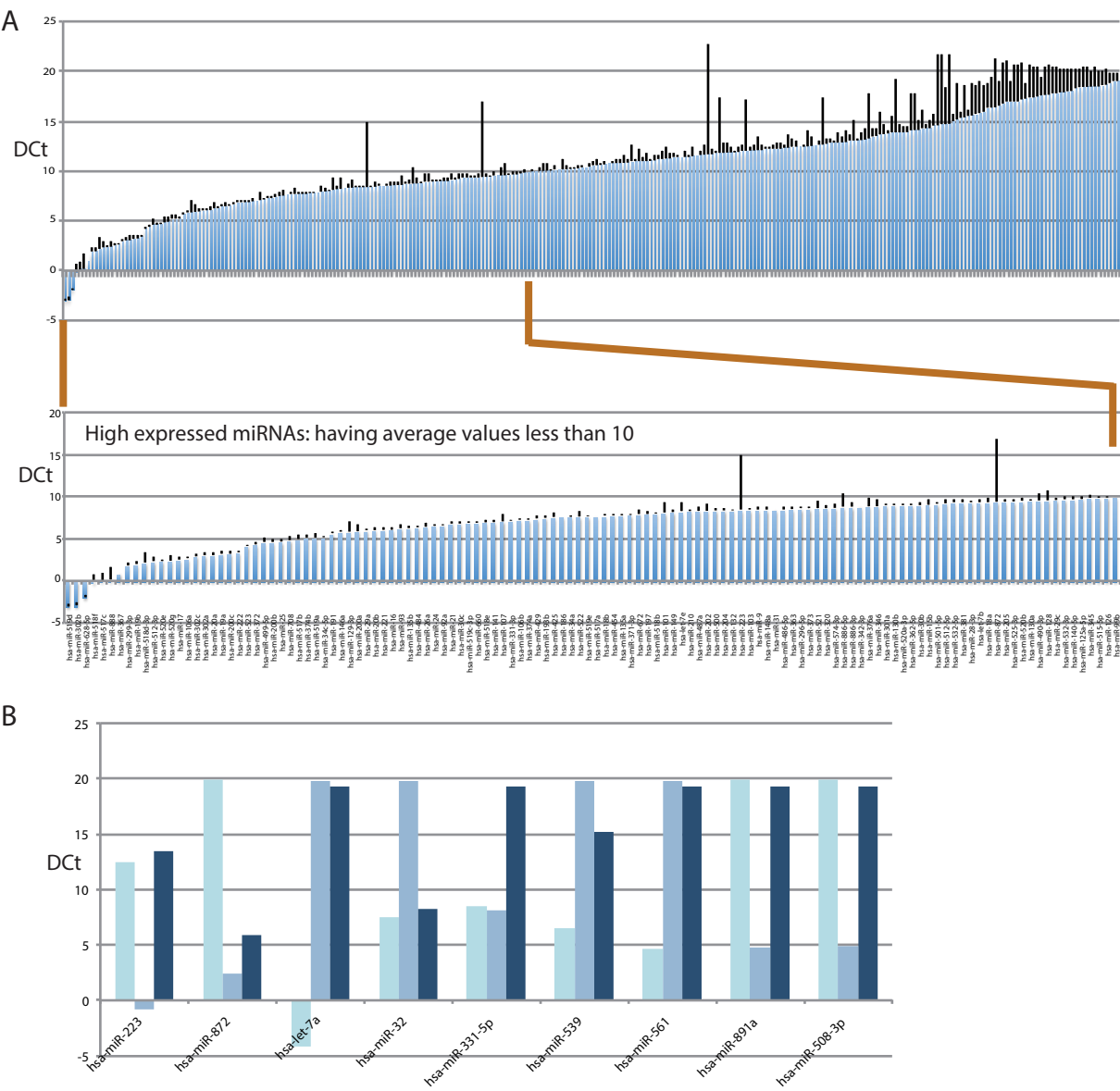

Fig. S3 Siti et al.

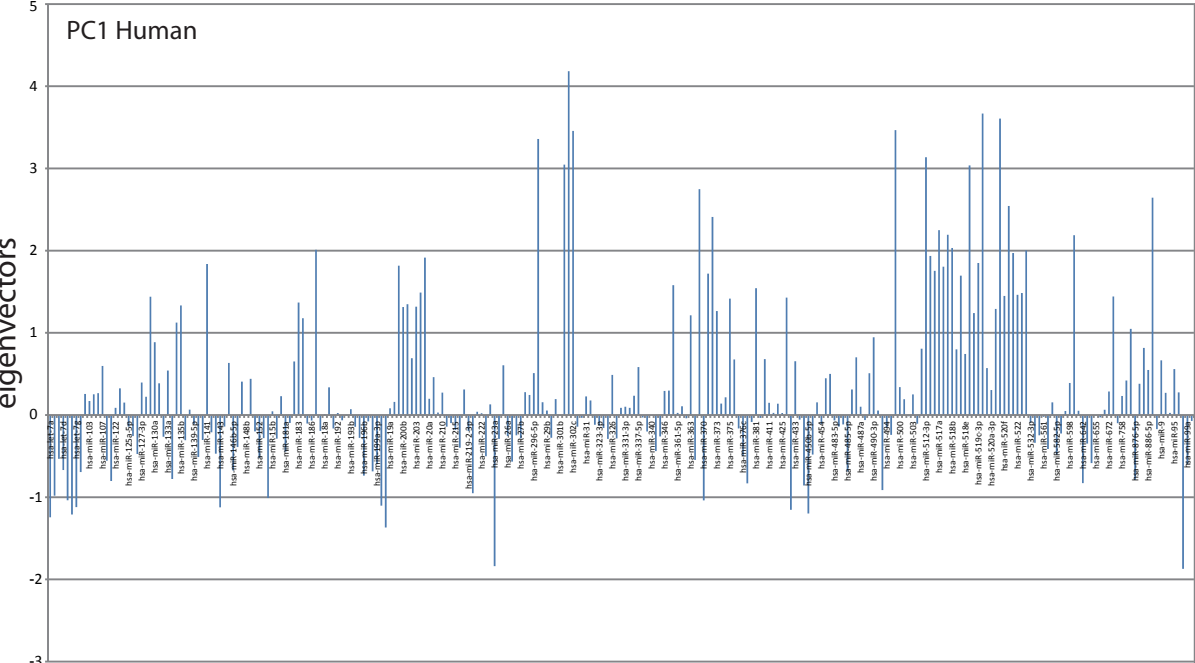

Fig. S4 Siti et al.

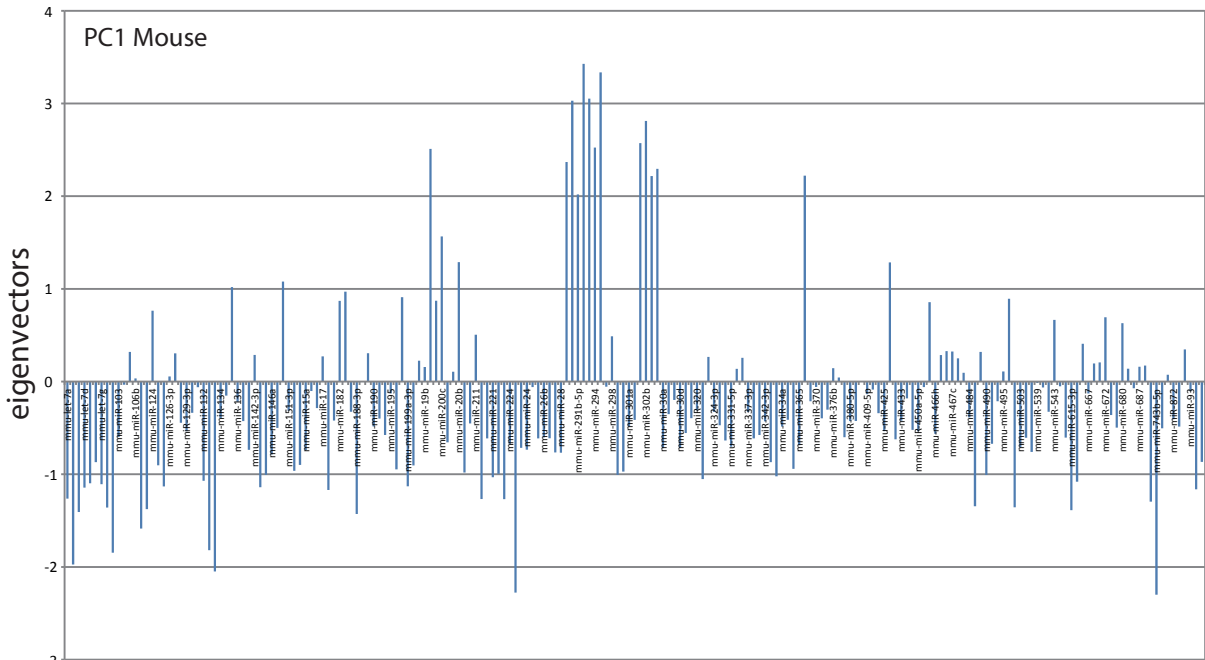

Fig. S5 Siti et al.

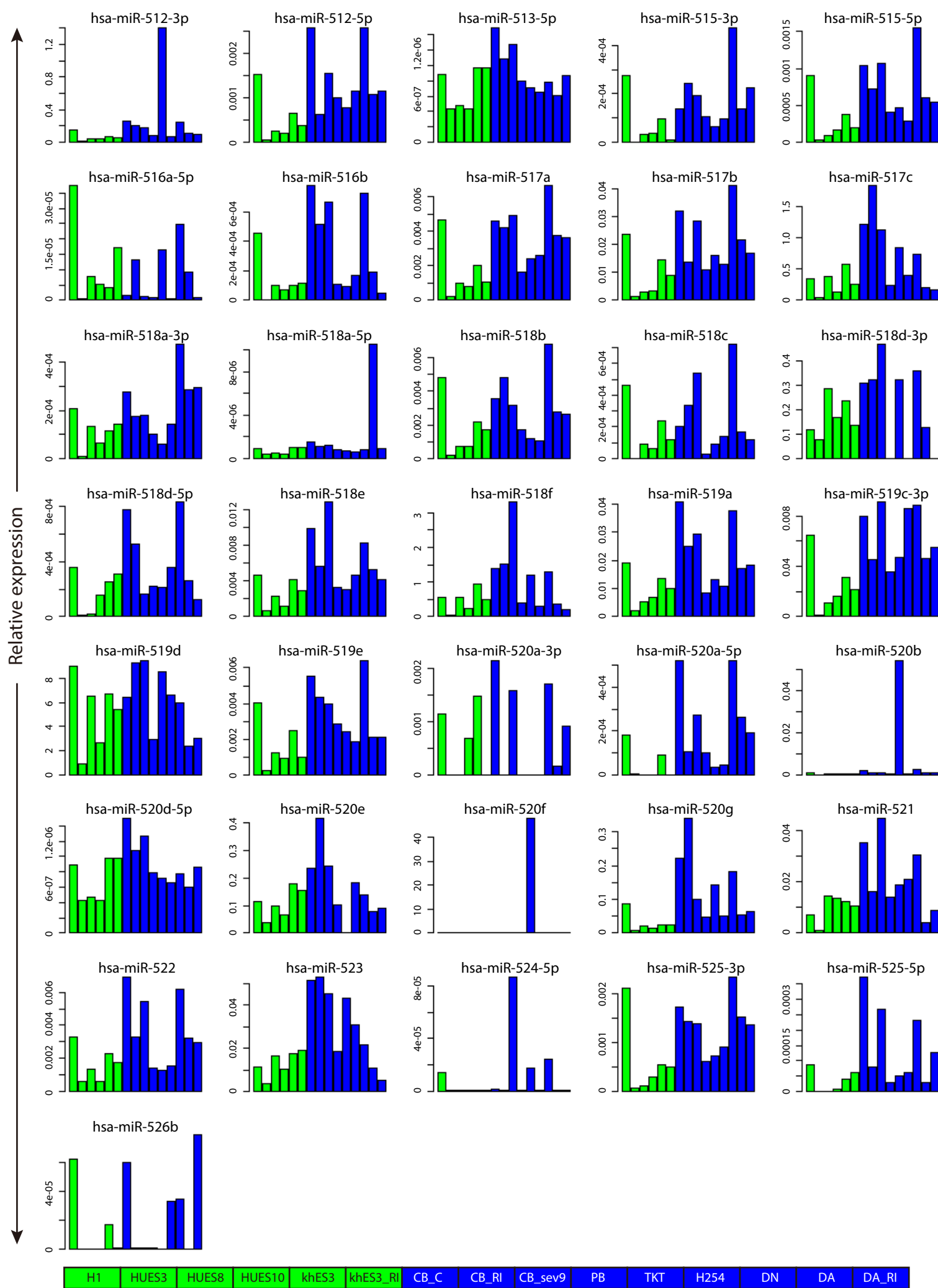

Supplement: File S1 — Figure S1. Expression pattern of miRNA of purified and un-purified iPS. Expression of miRNA was examined by qPCR array usingcDNA prepared from purified iPS cells and un-purified iPS cells, which are mixture with feeder cells. Average values are average DCt values of all human iPS. Figure S2. Expression pattern of miRNAs of purified human iPS, tkCB 7-4 was analyzed 3 times independently. Lower panel in A is enlarged part of upper panel with name of miRNA. There are 9 miRNAs which have more than 5 SD value in all, and row data of 3 samples of these miRNAs are shown in B. Figure S3. The eigenvectors of the first component of PCA of human cells. Figure S4. The eigenvectors of the first component of PCA of mouse cells. Figure S5. Relative expression values of members of C19MC miRNA. Expression levels of members of C19MC miRNA of human ES (green bar) and human iPS (blue bar) are shown. (PDF) [file pone.0073532.s001.pdf]
